# Growth parameters of NICU admitted low birth weight preterm neonates at corrected ages of 6 and 12 month

**Published:** 2012-09

**Authors:** Zia Islami, Razieh Fallah, Toktam Mosavian, Mohammad Reza Pahlavanzadeh

**Affiliations:** 1 *Department of Pediatrics, Shahid Sadoughi University of Medical Sciences, Yazd, Iran.*; 2 *Department of Pediatrics, Children Growth Disorders Research Center, Shahid Sadoughi University of Medical Sciences, Yazd, Iran.*; 3 *Department of Laboratory Sciences, Shahid Sadoughi University of Medical Sciences, Yazd, Iran.*

**Keywords:** *Low birth weight*, *Growth disorders*, *Weight*, *Height*

## Abstract

**Background: **Low birth weight (LBW or birth weight <2500 g) is one of the most serious health problems in today's world.

**Objective:** The purpose of this study was to evaluate growth parameters of neonatal intensive care unit (NICU) admitted LBW preterm neonates at corrected ages of 6 and 12 months.

**Materials and Methods:** In a prospective cohort (follow up) study, all LBW preterm neonates whom were admitted to Shahid Sadoughi Hospital NICU in 2008, followed up for one year and their weight, height and head circumference evaluated at ages of 6 and 12 months.

**Results:** Twenty four boys and 26 girls with mean gestational age of 31.3±2.7 weeks and mean birth weight of 1480.3±422.8gr were evaluated. The most frequency of underweight and short stature was seen at the age of six months. Exclusive breast feeding infants had the lowest frequency of underweight at the age of six. Frequency of underweight at the age of six months and NICU stay days were more in neonates with birth weight of less than 1000 gr.

**Conclusion:** It is necessary to emphasize on the importance of growth assessment of LBW children and proper education of their mothers about nutrition of their children for early and timely diagnosis and management of growth retardation and prevention of subsequent problems.

## Introduction

‘‘Low birth weight (LBW or birth weight of less than 2500 gr) is one of the main determinants of neonatal and postnatal morbidity. LBW neonates are sub grouped according to the first weight determination after birth: moderately low birth weight (MLBW): between 1500 and 2499 gr, very low birth weight (VLBW): less than 1500 gr and extremely low birth weight (ELBW): less than 1000 gr (1)”. 

According to World health organization (WHO) statistics, the rate of LBW is 17% in the whole world (6% in industrialized countries, 21% in developing countries and it is 10% in Islamic Republic of Iran) (2). Based on the result of one study, the LBW rate in Yazd, central city of Iran, is 8.4% (3).

Over the past two decades, LBW rate has increased primarily because of an increase in preterm births (4). LBW and prematurity are the second leading causes of infant mortality after congenital anomalies, but contribute disproportionately to the infant mortality rate (deaths in the first year after birth) (5). “On the other hand, infant mortality rate in LBWs are 40 times more than infants with normal birth weight (NBW or 2500-4000 gr)” (6, 7). 

Up to the age of 15 years, LBW children showed a higher risk of death and in LBW children, rate of hospitalization was higher for the first 14 years of life (8). By increase in the number of neonatal intensive care unit (NICU) and promotion in the equipment and increase in the number of preterm and LBW neonates, some concerns exist about growth outcome of such children. In 2010, the centers for disease control and prevention (CDC) recommended that the WHO growth charts be used for children of less than two years, and the CDC growth charts for children aged 2-19 years (9). 

In growth monitoring of preterm infants by standard growth charts, gestational age should be corrected for weight through 24 months of age. It is through 40 months of age for stature and 18 months of age for head circumference (10). 

Little is known about the growth of LBW infants in Iran. The purpose of this study was to evaluate growth parameters (weight, height and head circumference) of NICU admitted LBW preterm neonates at corrected ages of six and 12 months in Yazd, I.R.Iran. 

## Materials and methods

The sample size based on Z formula and a confidence interval of 95% with 80% power to detect a significant difference between the two groups with a level of 0.05 was calculated to be 50 children. Gestational age less than 37 weeks was considered as preterm neonate. In a prospective cohort (follow up) study, all consecutive preterm LBW neonates whom were admitted to Shahid Sadoughi Hospital NICU in 2008, followed up and their growth parameters (weight, height and head circumference) in corrected ages of 6 and 12 months were evaluated. Corrected age was calculated by subtracting gestational age from 37 weeks and the result was subtracted from chronological age: (37-gestational age= A, Corrected age= Chronological age- A).

Multiple pregnancies, severe asphyxia, children with major congenital malformations, small for gestational age (SGA), chromosomal abnormalities, genetic syndromes and neonates who had serious complications such as intraventricular hemorrhage, patent ductus arteriosus, hydrocephaly, bronchopulmonary dysplasia and necrotizing enterocolitis during NICU admission period were excluded. 

All babies were weighted by children digital weighing scale with sensitivity of 10 gram without diapers. The weighting scale was calibrated at regular intervals. The supine crown heel length was measured on the infantometer with the help of an assistant to the nearest millimeter in the recumbent position.

The weighing scale and infantometer were Germany made Seca. Head circumference was measured using a flexible non-stretchable tape measure which runs from the supraorbital ridge to the occiput in the path as the maximum occipitofrontal circumference. To obviate errors due to interobsever variations, all measurement were made in Pediatric Clinic of Shahid Sadoughi Hospital by one pediatric resident involved in this research. For assessment of growth parameters, the WHO growth charts were used (11).

Weight below the third percentile on a standard growth curve weight, as reached 40 postmenstrual weeks, was considered underweight. Height of less than two standard deviation from height age was considered short stature (11) and head circumference of more than two standard deviation below the mean for a given age, sex, and gestation was considered microcephaly based on definition of the American academy of neurology (AAN) practice parameter (12).

The same children were evaluated at six months, followed up to 12 months and their growth parameters were evaluated again at 12 months. This study was approved by the Ethic Committee of Shahid Sadoughi University of Medical Sciences, Yazd, Iran. 

**Statistical analysis**

The data were analyzed using SPSS (Statistical Packages for Social Sciences) version 15 statistical software. Chi-square test or Fisher exact test were used for data analysis of qualitative variables and mean values were compared using independent T-test. Differences were considered significant at p-values of less than 0.05.

## Results

Parents of five infants did not return for follow up and therefore their infants were excluded from the study. Twenty four boys (48%) and 26 girls (52%) with mean gestational age of 31.3±2.7 weeks (range=26-37 weeks) and mean birth weight of 1480.3±422.8 gr (range= 700-2300 gr) were evaluated. Comparison of mean weight, height and head circumference at birth and gestational age based on sex is presented in table I and indicates that mean of growth parameters was not statistically significant different in both sexes. One girl died at the age of four months due to aspiration pneumonia and one boy died at 9 months due to sepsis.

Frequency of underweight, short stature and microcephaly at ages of six and 12 is shown in table II which indicates that underweight (18.4% at the age of six months vs. 6.2% at the age of 12 months, p=0.02) and short stature (34.7% at the age of six months vs. 6.2% at the age of 12 months, p=0.001) were more prevalent at the age of six month. Comparison of frequency of underweight, short stature and microcephaly at the ages of six and 12 months based on nutrition is presented in table III that indicates underweight at the age of six months [6.25% (1 of 16 infants) in exclusive breast milk feeding vs. 21% (8 of 38 infants) in mixed and only bottle feeding, p=0.03] was more frequent in bottle fed infants. 

Comparison of frequency of underweight, short stature and microcephaly at ages of six and 12 months based on sex is presented in table IV which indicates frequency of growth disorders in six and 12 months were not statistically different in girls and boys. Comparison of frequency of underweight, short stature and microcephaly at the ages of six and 12 months and mean of NICU stay days based on birth weight are shown in table V that indicates frequency of underweight at the age of six months (p=0.009) and NICU stay days was more in neonates with birth weight of less than 1000gr (p=0.001). 

**Table I:** Comparison of mean of weight, height and head circumference at birth and gestational age based on sex

**Data**	**Girls** **Mean ±SD**	**Boys** **Mean ±SD**	**p-value**
Weight in grams	1509.6 ± 45.8	1458.8 ± 38.9	0.67
Height in centimeter	39.7 ± 3.8	39.1 ± 3.9	0.56
Head circumference in centimeter	28.2 ± 2.4	27.8 ± 2.3	0.51
Gestational age in weeks	31.7 ± 3.1	30.9 ± 2.4	0.33

**Table II:** Frequency of underweight, short stature and microcephaly at 6 and 12 months of age

**Data**		**At 6 months** **n (%)**	**At 12 months** **n (%)**	**p-value**
Weight				
	Underweight	9 (18.4%)	3 (6.2%)	0.02
	Normal	40 (81.6%)	45 (93.8%)	
Height				
	Short stature	17 (34.7%)	3 (6.2%)	0.001
	Normal	32 (65.3%)	45 (93.8%)	
Head circumference				
	Microcephaly	4 (8.2%)	0 (0%)	0.76
	Normal	45 (91.8%)	48 (100%)	

**Table III:** Comparison of underweight, short stature and microcephaly at the ages of six and 12 months based on nutrition

** Nutrition**		**Breast milk**	**Formula**	**Mixed**	**p-value**
**Data**					
Weight in 6 months					
	Underweight	1	3	5	0.03
	Normal	15	10	15	
Weight in 12 months					
	Underweight	0	1	2	0.35
	Normal	15	11	19	
Height in 6 months					
	Short stature	5	5	7	0.92
	Normal	11	8	13	
Height in12 months					
	Short stature	1	0	2	0.35
	Normal	14	12	19	
HC in 6 months					
	Microcephaly	1	2	1	0.59
	Normal	15	11	19	
HC in 12 months					
	Microcephaly	0	0	0	-
	Normal	15	12	21	

HC: Head circumference.

**Table IV:** Comparison of underweight, short stature and microcephaly at the ages of six and 12 months based on sex

** Sex**		**Girl**	**Boy**	**p-value**
**Data**				
Weight in 6 months				
	Underweight	5	4	0.76
	Normal	20	20	
Weight in 12 months				
	Underweight	3	0	0.3
	Normal	22	23	
Height in 6 months				
	Short stature	6	11	0.25
	Normal	19	13	
Height in 12 months				
	Short stature	1	2	0.75
	Normal	24	21	
HC in 6 months				
	Microcephaly	3	1	0.1
	Normal	22	23	
HC in 12 months				
	Microcephaly	0	0	-
	Normal	25	23	

HC: Head circumference.

**Table V:** Comparison of underweight, short stature and microcephaly at the ages of six and 12 months and mean of NICU stay days based on birth weight

** Birth weight**		**<1500gr**	**1000-1499gr**	**1500-2499gr**	**p-value**
**Data**					
Weight in 6 months					
	Underweight	5	2	2	0.009
	Normal	5	11	24	
Weight in 12 months					
	Underweight	1	1	1	0.22
	Normal	9	11	25	
Height in 6 months					
	Short stature	5	4	8	0.28
	Normal	5	9	18	
Height in12 months					
	Short stature	1	0	2	0.14
	Normal	9	12	24	
HC in 6 months					
	Microcephaly	1	1	2	0.68
	Normal	9	11	24	
HC in 12 months					
	Microcephaly	0	0	0	-
	Normal	10	12	26	
Stay days in NICU (mean±SD)		48.5 ± 16.3	31.4 ± 14.1	15.6 ± 6.6	0.001

HC: Head circumference.

## Discussion

Results of this study showed that growth parameters of LBW children partially improved at the age of one year as frequency of underweight and short stature decreased and no child had microcephaly in one year. In an Indian study, children with birth weight of less than 1.25 kg and less than 30 gestation weeks had delayed and poor catch up growth and they had considerable lag that persisted up to 1 year of age (13).

In a Chinese study, growth parameters of 132 LBW neonates and 49 normal birth weight term infants were followed up and compared. LBW children had lower weight, length, and head circumference from birth for up to three years (14). A prospective cohort study in South Africa which was conducted on 139 VLBW (birth weight less than 1500 gr) infants, showed that VLBW had poor growth initially which followed by gradual catch up growth, but deficits in length for age persisted at 20 months postmenstrual age compared with healthy term infants (15).

Evaluation of growth in LBW infants should be more emphasized since growth failure in such infants might be associated with many complications including increasing the number of hospitalization, learning disabilities, growth retardation in childhood and reductions in adult lung function and capacity (16, 17).

In the present study, infants who had exclusive breast feeding for six months had lower frequency of underweight at the age of six months and it is in compliance with other studies which suggested a beneficial effect of breastfeeding on childhood growth rates of LBW newborns and efforts must be continued to breast feed all low birth weight neonates in the NICU and even after discharge (18-20).

In this study, growth parameters of LBW infants partially improved in year one and it might be due to emphasize to continue breast milk feeding, education of supplementary feeding and evaluation of physical growth of these high risk babies by researchers and personnel of health centers. “In a study in Norway, 118 VLBW infants had significant catch-up growth in weight and length during the first year. But, their head circumference showed a normal growth pattern after two months” (21).

In a study in São Paulo of Brazil, growth patterns of 60 neonates weighing less than or equal to 2000 gr during the first year were followed up. It showed that in spite of their corrected age, such infants growth curves were below the reference curve standards (22).

Limitations of the present study were; small sample size, lack of a control group and short follow up period. Therefore, it is suggested that further studies be conducted with a larger sample size and a control group (normal birth weight infants) for comparison. In conclusion, physical growth assessment is necessary in low birth weight infants and accurate recording of growth parameters of LBW infants by personnel of health centers, regular and frequent visiting of such children, education of their parents about growth process, and follow up and encouragement to exclusive breastfeeding should be emphasized for early and timely diagnosis, investigation and management of growth disorders in LBW children.

Factors influencing catch up growth should be recognized and adequate measures to improve growth parameters (like attention to feeding practices) and overall outcome of such children should be taken. Therefore, it is significant to curb the rate of LBW neonate and to offer scientific guidance on care and early training to promote development and growth of infant.

## Footnote

***This article was extracted from residential thesis.***
